# Streamlining and improving controlled drug dispensary workload in a London teaching hospital following the implementation of automated dispensing cabinets on wards: A quality improvement project^☆^

**DOI:** 10.1016/j.rcsop.2025.100594

**Published:** 2025-03-23

**Authors:** Áine Walsh, Emma Jeffrey, Kit Lai

**Affiliations:** King's College Hospital, United Kingdom

**Keywords:** Automated dispensing cabinets, Inventory management, Controlled drugs, CDs, Periodic automated replenishment, Automated ordering

## Abstract

An efficiently run pharmacy dispensary has the potential to positively impact patients stay in hospital. Pharmacy dispensary providers work in a fast-paced, hectic environment and are finding it increasingly difficult to keep up with workload demands. In order to meet the increasing demand on resources, pharmacy teams are incorporating Quality Improvement (QI) initiatives to improve efficiencies within systems for the purpose of freeing up nursing and pharmacy staff time, improve patient flow and improve patient safety. This QI project sought to develop a standardised inventory control formula for controlled drugs (CDs) stocked in ADCs in clinical locations in order to reduce the frequency of CD stock orders, minimise the requirement for manual nurse generated orders and in doing so streamline workload for dispensaries whilst ensuring sufficient CD stock holding levels in clinical locations. The introduction of ADCs corresponded with an increase in dispensary workload at the main hospital site at King's College Hospital (KCH). Retrospective time series analysis of the monthly CD dispensing data was analysed for Surgical and Trauma wards over a 27-month period using the Statistical Control Process (SPC) tool. Volume of stock CD dispensing transactions, volume of stock Vs non-stock CD dispensing transactions, volume of weekday vs weekend, morning, afternoon and out of hours CD stock workload were measured. Two interventions were implemented: (i) stock optimisation informed by the utilisation of CD dispensing data issued from the ADC in central pharmacy; (ii) stocklist rationalisation to ensure the most commonly used CDs were added to CD stocklists and the least commonly used CDs were removed from stocklists in addition to the development of a standardised inventory management formula. The main outcome measure was the volume of stock CD dispensing transactions processed by central pharmacy. Secondary outcome measures included the volume of stock CD dispensing transactions at weekends Vs weekdays in addition to the split in workload between mornings, afternoons and out of hours. A reduction in stock CD workload for Surgical and Trauma wards was demonstrated following the stock optimisation review whereas this was not significantly impacted by the stock rationalisation or application of the standardised inventory management formula. Weekend workload reduced by 30 % in comparison to pre-ADC baseline period. Morning, afternoon and out of hours CD stock workload demonstrated a sustained improvement following stock optimisation, stock rationalisation and following the application of the standardised inventory management formula. The only exception to this sustained improvement was in October 2023 following the implementation of a new Trust wide EMPA system. The perfect formula to determine CD stock inventory levels remains elusive, however, as nurses are still required to order stock manually via CD requisition books for stock CD requests. Continual cycles of reviews are made possible through improved ease of data availability with the use of ADCs. Suggestions to further improve the formula are explored although not tested as part of this QI. The proposed improvements to the standardised formula together with more widespread application to different specialties is recommended as the next steps to this QI.

## 1 Problem

The primary function of a hospital pharmacy service is the provision of a safe and efficient supply of medications for patients. The NHS is coming under increasing financial strain. Inpatient activity in the NHS has seen an average 3 % year-on-year increase between 2013 and 2020; while staff vacancy rates remain stubbornly high at 111,000 which was recorded in Q3 of 23/24.^1^^,^^2^   This has culminated in staff having to deliver more with less; the pharmacy being no exception. In a 2023 NHS survey, 73.8 % of staff reported experiencing unrealistic time pressures whilst 30.4 % of staff surveyed reported burnout.^2^ The demands on the supply of medications have increased, and so too have the frequency of supply disruptions exacerbated by Brexit and the global COVID-19 pandemic. Pharmacy departments need to consider new and innovative ways to create capacity to withstand the increasing demand for resources.^3^  .

Adopting technology in healthcare settings to automate processes, improve efficiencies, and improve patient safety is becoming increasingly common. ^4^ King's College Hospital (KCH) has recently implemented Automated Dispensing Cabinets (ADCs) for the storage of medicines in inpatient clinical locations. ADCs are one form of technology that has been used in healthcare settings since the 1990s.^5^ The benefits of ADCs have been broadly described in the literature to improve efficiencies, improve patient safety, and reduce cost.^6^ That said, there is a paucity of studies that specifically focus on whether the storage of controlled drugs (CDs) in ADCs can improve efficiencies.

The safe and secure handling and storage of controlled drugs (CDs) is stipulated by the Misuse of Drugs Act 1971.^7^ Accurate and up-to-date record keeping for all CD transactions is a compulsory requirement to ensure staff are adhering to the governing policies. Such activities are time-consuming and can detract from nursing and pharmacy time spent on patient-facing activities. A recent study from Nature concluded that 21 % of a nursing shift is spent on documentation processes.^8^ The same study concludes how the use of technology can reduce the need to multitask; improving patient safety as a result.

Efficiency savings have been demonstrated when ADCs are utilised for the storage of CDs but only when the use of the electronic CD register is adopted.^9^^,^^10^ These studies focus predominantly on the medication administration process while there is little to no focus on the current inefficiencies around the ordering and dispensing process. Predating the use of ADCs, CD inventory levels were solely monitored and managed by nursing staff which in the current climate of nursing shortages is becoming more and more challenging. When inventory control is poor, increased pressure is put on the dispensary team to reactively process and dispense medications urgently. The more inventory locations where this is the case, the more exaggerated the problem in the pharmacy becomes culminating ultimately in delayed doses and potential patient safety implications.^11^  There is a huge variation in how hospital pharmacy dispensaries operate worldwide. Some hospitals provide a seven-day service, some operate under a centralised model with one central dispensary and others adopt the use of satellite pharmacies also known as a decentralised model.^12^ Regardless of the service model of delivery, optimising systems use is crucial to enable the realisation of associated efficiency benefits.

The pharmacy dispensary team have reported an increase in the volume of stock CD workload in areas that have had ADCs introduced. This was picked up through routine activity and performance metrics reporting that warranted further investigation which is the basis of this Quality Improvement (QI) project. Since the introduction of ADCs, the occurrence of manual ordering remains. Determining optimum inventory levels is essential to ensuring sufficient stock holding in clinical areas minimising the frequency of orders generated. Making efficiency savings in dispensary by reducing the frequency of CD orders ultimately facilitates quicker turnaround times for workload overall; something which has proven to have a direct impact on patient care and patient flow in hospitals.^13^  .

## 2 Background

At KCH, the main dispensary located at Denmark Hill has utilised ADCs in the pharmacy to support the management of CDs since 2010. The pharmacy ADC secures all CD stocks held in the department with all transactions recorded electronically in the register providing a full audit trail. Nurses have traditionally ordered controlled drugs during quieter periods at weekends and out of hours which is often prompted by the daily CD count. Weekend and out-of-hours ordering poses a particular challenge to pharmacy due to the limited availability of staff working during these periods. Nursing staff are also, quite often, restricted from ordering high volumes of stock due to space constraints and therefore clinical areas which have a high CD demand, for example, surgical and trauma wards, are required to visit the pharmacy more regularly to obtain the required stock.

The installation of 58 profiled ADCs in clinical ward locations at the main hospital site in 2023 provided an opportunity to automate the ordering and restocking process of CDs for clinical areas from the pharmacy. Cabinets were interfaced with the trust EPMA system. Stock CDs traditionally stored in secure CD cabinets located in clinical treatment rooms were moved into the lidded bins within the ADC on wards that then required the authorisation of two registered, clinical staff members to access. Non-stock and patients' CDs remain stored in secure wall-mounted CD cabinets.

Prior to the introduction of ADCs, CDs were requested by authorised nursing staff via a CD requisition book. Requisition books can be taken to the pharmacy at any time of day during departmental dispensary opening hours (09:00 a.m. until 19:30 p.m. Monday through Friday and from 09:00 a.m. until 17:00 p.m. weekends and bank holidays) or out of hours in an emergency via the residency service, requested through the on-call pharmacist. This service is intended for the provision of advice and the emergency supply of medications only.

A change to the documentation process occurred with the implementation of ADCs. Automatically generated orders were received via email, printed and signed by dispensary staff when items were supplied. Nursing staff received and signed this printout and are then expected to file the copy for two years in accordance with legal requirements and trust policy.

The Periodic Automated Replenishment (PAR) level is the maximum level set for each drug stored within an ADC, the re-order level, once reached, is the level at which the system triggers an order. The critical level, once reached, also triggers an order but crucially is set to a lower level than the re-order level. For CDs at KCH, following the installation of ADCs, an order is automatically generated Monday through Friday once the re-order level is reached. This order is sent once per day at 6:00 a.m. to a shared pharmacy email inbox for dispensary staff to process. At weekends an order is also automatically triggered once the critical level is reached and an order is sent at 6:00 a.m. The critical level is set at a higher threshold than the re-order level making CD orders at weekends less likely. The system administrator is a specialist role within the pharmacy team responsible for setting and amending inventory levels for each drug. In the absence of any published literature on the topic of setting inventory levels for CDs stocked in ADCs, at implementation, a combination of anecdotical advice from the suppliers and feedback from the nursing staff was followed. The re-order and critical levels were set for each drug based on a set formula defined at the beginning of implementation; re-order levels were set at 50 % of the PAR and the critical level was set at 50 % of the re-order level.

Codeine was changed to a Schedule 2 medication at KCH in April 2023. For this reason, codeine was excluded from the data set. Stationary; CD requisition books and registers were also excluded from the data.

**Fig. 1:**
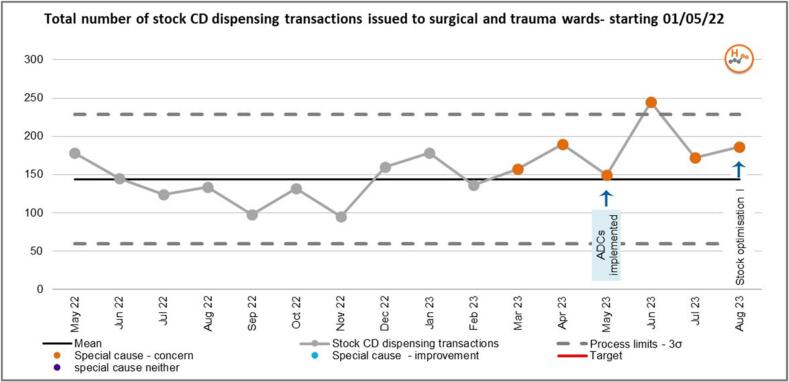
SPC chart showing the total number of stock CD dispensing transactions for surgical and trauma wards in the baseline period pre-ADC implementation (May 2022–April 2023) and in the period following implementation.

There are five surgical and trauma wards at the main hospital site at Denmark Hill with an average of 21 beds per ward. ADCs were installed on these wards between March and May of 2023. An increase in CD dispensing workload was observed coinciding with the installation of ADCs in these clinical locations. This was identified through the monitoring of key performance indicators (KPIs) and activity metrics ordinarily tracked by the dispensary team. Data was inputted into a statistical process chart (SPC) tool to determine whether the shift in activity was due to chance.^14^ The astronomical data point outside the Upper Control limit (UCL) in June 2023 is indicative of a system that is out of control and thus requires an intervention (Fig. 1.)

**Fig. 2:**
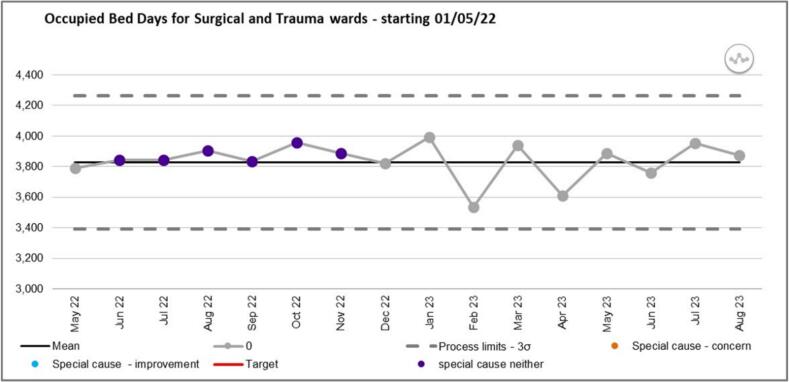
Occupied Bed Days for all five surgical and trauma wards during the initial study period (May 2022 until August 2023).

The cumulative activity on all five surgical and trauma wards across the same period, in terms of occupied bed days (OBDs), was also explored to exclude causality between an increase in OBDs with increased CD dispensing workload (Fig. 2). No special cause variation was noted in activity therefore ruling this out as a contributing factor for the observed increased workload.

The overall aim of this QI was to reduce the frequency of automated CD orders generated, whilst ensuring sufficient CD stockholding on surgical and trauma units. Secondary aims included a reduction in the generation of orders for stock CDs at weekends. This would be achieved through the establishment of standardised formulae to determine the optimum, PAR, critical and reorder levels (ROLs) for all CDs using CD consumption data for surgical and trauma ADCs.

## 3 Design and strategy

A continuous QI project with a prospective pre- and post-implementation design to evaluate CD dispensing transaction data following the introduction of ADCs. Surgical and trauma wards were chosen because the highest overall increase in workload was observed in this speciality. Surgical wards also have a wide variety of CDs allowing for any standardised formula applied to be tested across a wide range of products and formulations. ADCs were installed across all surgical and trauma wards between March and May 2023, allowing for three months of transaction data collection when the first optimisation took place in August 2023.

ADC stock optimisation, in the context of this QI, is defined as the review of CD inventory stock levels stored within the ADC to improve operational efficiency related to CD ordering and restock functions. ADCs were optimised by multiple, iterative interventions that have been summarised in Table 1.

**Table 1:** Summary of interventions.

Interventions/baseline	Specialty	No of locations	Period	Summary of intervention
Baseline	Surgical & Trauma wards	5	May 2022–August 2023	ADCs were installed in all Surgical and Trauma wards at the main Hospital site at Denmark Hill during the period from March 2023 until May 2023
Stock optimisation I	Surgical & Trauma wards	5	August 2023	PAR, *Re*-order, and critical levels were reviewed after 3 months of dispensing data from the central pharmacy
Stocklist rationalisation/ stock optimisation II	Surgical & Trauma wards	5	March 2024	All CDs supplied to the 5 surgical and trauma wards were reviewed to determine what to add/remove from stocklists based on usage over 90 days. PAR, *Re*-order and critical levels were calculated by 3 months of usage data

Dose administration data was utilised to inform stock level optimisation to influence a change in practice.

Stock Optimisation I:

The first intervention occurred in August 2023 and involved a review of all stock CD orders for all surgical and trauma wards at the main hospital site at KCH three months following the installation of ADCs. This review was undertaken by two senior pharmacy technicians working in inpatient services. Optimisation involved increasing or reducing PAR, re-order and or critical levels for each CD stock line based on the frequency with which these were issued from the pharmacy within the previous three-month period (May–July 2023). PAR levels were increased for items issued most often and reduced for items issued infrequently by the system administrator. *Re*-order and critical levels were set in the same way as they had been set for going live. Re-order levels are set at 50 % of the PAR and critical levels are set at 50 % of re-order levels. Inventory levels were amended by the system administrator.

Stock rationalisation/optimisation II:

The second intervention was carried out in March 2024 allowing a six-month washout period following the first intervention. Stock rationalisation involved a review of all CDs, both stock and non-stock, issued to the five surgical and trauma wards over eight months (May-Dec 2023), to curate the most optimal stock lists for each location. Specifically, this involved reviewing the dispensing issue data from the CD ADC located in the central pharmacy to the surgical and trauma wards over this period. The data was accessed via the reporting software system MedX™ which is the platform the ADC company Omnicell™ utilise to host and allow users to access transaction data. All stocklist items were reviewed by the project implementation lead and if the items were dispensed on average once or more per month, these items were to remain on the stocklist; if the items were dispensed less than once per month a recommendation was proposed for these items to be removed from stocklists. Similarly for non-stock items, if the items were issued more than once per month, a recommendation was proposed for these items to be added to the respective stocklists. A list of recommendations was compiled and sent to the principal pharmacist for surgery and trauma to either accept or reject the changes. Matrons and nursing team leads were also consulted and the proposed amendments to the stocklists were either accepted or rejected. Justifications were required for all recommendations which were rejected. The stock lists were then amended accordingly. CDs removed from stocklists were physically removed from the ADCs and returned to the dispensary by a senior technician by trust procedures before the items were deleted by the system administrator. The items needed to be deleted on the same day as they were removed, otherwise, the ADCs would have triggered a stock order the following day to replenish PAR levels.

**Fig. 3:**
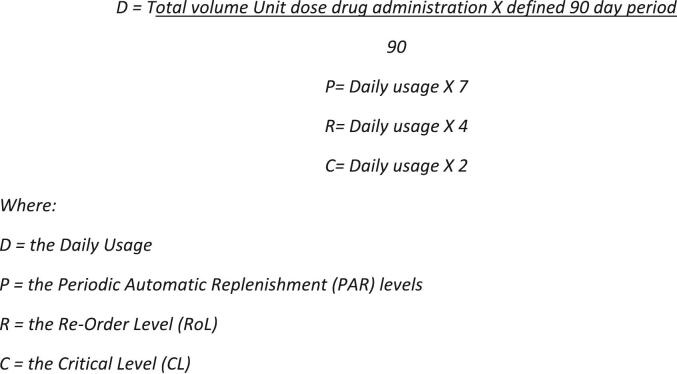
Calculation of inventory levels.

The second stock optimisation review was also undertaken in March 2024 by the project implementation lead. Dose administration data retrieved from MedX™ for each CD, on each ward was compiled over 90 days using the pivot table function in Microsoft Excel. This was based on unit dose drug administration data i.e. doses administered to patients in beds from January–March 2023. The cumulative administration data for each drug, unique to each ward, was contemporaneously divided. This generated the average daily usage for each drug, for each ward. PAR levels were set based on a seven-day stockholding requirement rounded up to the nearest pack plus half a pack size. This is a requirement dictated by how the Omnicell™ system generates orders. The system does helpfully alert the system administrator to this if not done by the system's requirements. A week's stockholding was determined whilst accounting for space constraints in the ADCs. The PAR, re-order and critical levels were calculated by multiplying the daily usage by 7, 4, and 2 respectively and then finally rounded up to the nearest pack size plus half a pack in Microsoft Excel (Fig. 3). *Re*-order levels were based on four-day stockholding to ensure sufficient inventory over weekends and bank holidays, preventing unnecessary ordering and avoiding stockouts. The critical level function was utilised to minimise the generation of automated orders at weekends. At weekends, the system is programmed to order based on critical levels as opposed to re-order levels. Critical levels were utilised from go-live but were optimised as part of this QI. The purpose of optimising critical levels was to further minimise the auto-generation of unnecessary orders at weekends while maintaining sufficient stock inventory levels. The analysis was carried out by the project implementation lead and inventory changes were made by the system administrator.

### 3.1 Description of QI measures

Acknowledging that there are normal variations in volumes of CD dispensing over time, SPC charts were used for the statistical interpretation of the data.^14^  .

Specifically, SPC enables the identification of the following patterns where:

• Special cause variation comes from outside of the system and causes recognisable patterns that may present as a shift, trend or astronomical point in the data. This can either be good or bad and is depicted as a special cause improvement or special cause concern on the SPC chart
• A shift is defined as a run of 6 or more data points above or below the mean
• A trend is defined as a run of 6 or more data points all consecutively ascending or descending which may or may not cross the mean
• Upper Control Limit (UCL) and Lower Control Limit (LCL) are two horizontal lines on the control chart which represent the maximum acceptable variation from the process mean. When data points fall within the control limits, the process is considered as being stable and in control. Data points that fall outside of the control limits usually require an intervention
• An astronomical point is defined as any data points that fall outside of the UCL or LCL
• Common cause variation demonstrates when a process is in control

The identification of any of the above trends would indicate that there is a change in process resulting in a change in outcome.

The primary outcome measure was to reduce the frequency of CD dispensing episodes across surgical and trauma wards. A secondary outcome measure was to reduce afternoon, out-of-hours and weekend CD dispensing workload by increasing the weekday morning workload. The following outcome measures were tracked for the different interventions applied across all surgical and trauma wards:

• Total number of stock CD dispensing transactions, where special cause improvement is measured (low being good)
• Stock CD dispensing transactions as a percentage of the total number of CD dispensing transactions (both stock and non-stock), where special cause improvement is measured (high being good)
• Morning stock CD dispensing transactions as a percentage of total CD stock workload, where special cause improvement is measured (high being good)
• Out-of-hours stock CD dispensing transactions as a percentage of total CD stock workload, where special cause improvement is measured (low being good)
• Total number of stock CD dispensing transactions at weekends, where special cause improvement is measured (low is good)
• Weekend stock CD dispensing transactions as a percentage of total weekly CD stock dispensing transactions, special cause improvement is measured (low is good)

Where an intervention has resulted in a sustained shift in data, a change to control limits was made and reflected in the SPC chart, otherwise, the control limits were kept the same across the intervention period.

The volume of out-of-hours requests for stock CDs was indicated as a balancing measure and monitored as a secondary outcome. This measured any unintended consequences of the interventions made.

## 4 Results

**Fig. 4:**
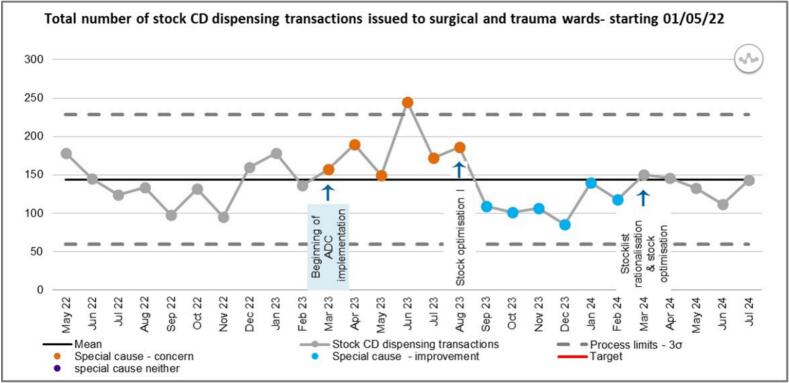
SPC chart showing total number of stock CD dispensing transactions for Surgical and Trauma wards.

Fig. 4 shows the total number of stock CD dispensing transactions for surgical and trauma wards. A run of six points above the mean (a shift) was observed from the beginning of ADC implementation in March 2023, representing a special cause of concern. A run of six points below the mean (a shift) was observed following the first intervention, representing a special cause improvement. A run of three points below the mean (a shift) was observed following the 2nd intervention- stock rationalisation/optimisation II predicting a special cause improvement in the future.

Table 2 summarises how there were 84 CDs in total across the five surgical and trauma wards. PAR levels were increased for a total of 24(29 %) products and decreased for 40(48 %). *Re*-order levels were increased for 6(7 %) of items and reduced for 56(67 %) of items. Critical levels were increased for 32(38 %) of items and reduced for 19(23 %) of items.

**Table 2:** Inventory level changes for stock optimisation I across all surgical and trauma wards.

***WARD***	***TOTAL CD STOCK LINES (%)***	***PAR INCREASED (%)***	***PAR DECREASED (%)***	***REORDER INCREASED (%)***	***REORDER DECREASED (%)***	***CRITICAL INCREASED (%)***	***CRITICAL DECREASED (%)***
*SURGICAL WARD 1*	*10*	*5*	*3*		*6*	*5*	*0*
*SURGICAL WARD 2*	*18*	*3*	*12*	*1*	*14*	*2*	*6*
*TRAUMA WARD*	*23*	*7*	*7*	*1*	*15*	*14*	*3*
*SURGICAL WARD 3*	*16*	*3*	*9*	*1*	*10*	*4*	*5*
*SURGICAL WARD 4*	*17*	*6*	*9*	*3*	*11*	*7*	*5*
*TOTAL*	*84* (100 %)	*24* *(29 %)*	*40* *(48 %)*	*6* *(7 %)*	*56* *(67 %)*	*32* *(38 %)*	*19* *(23 %)*

Fifty suggested changes to stock lines were proposed as part of the stock rationalisation review; 32(64 %) suggested removals from stocklists of which 24(75 %) were accepted and 18(36 %) proposed additions, 13(72 %) of which were accepted (Table 3). Reasons for the rejection of additions to stock lists included the strength of the product could be made up using multiple dose units of analogous existing stock products. Reasons for rejections for the removal of items were due to the requirement for formulations in certain patient types e.g. 10 mg/mL morphine sulphate ampoules needed to prepare subcutaneous preparations for end-of-life patients. Lower strength preparations were required for renally impaired patients as well as an item which was added to the newly updated hip fracture protocol therefore both deletions were rejected. Reasons for rejecting the addition of items included the following: oxycodone 20 mg/2 mL ampoules were rejected because the addition of oxycodone 10 mg/1 mL was accepted for the stocklist for surgical ward 1. Pregabalin 100 mg capsules were rejected because a dose could be prepared using a 25 mg plus a 75 mg strength capsule 75 mg capsule was already on the stock list for the trauma ward and the addition of the 25 mg capsule was accepted as part of this review. The addition of zopiclone was rejected suggesting the use of zolpidem instead. Zolpidem was not on the stocklist for surgical ward 3; and was therefore queried, the rejection was subsequently accepted. The buprenorphine 10 mg patch was rejected as a suggested addition to the stocklist for surgical ward 3 because it was deemed as not being required despite this product being dispensed eight times over the eight months thereby fitting the inclusion criteria for addition to the stocklist.

**Table 3:** Inventory level changes for stock optimisation II.

*WARD*	*TOTAL CD STOCK LINES (%)*	*PAR INCREASED (%)*	*PAR DECREASED (%)*	*RE-ORDERS INCREASED (%)*	*RE-ORDERS DECREASED (%)*	*CRITICAL INCREASED (%)*	*CRITICAL DECREASED (%)*
*SURGICAL WARD 1*	*11*	*0*	*5*	*2*	*3*	*0*	*8*
*SURGICAL WARD 2*	*11*	*7*	*3*	*7*	*1*	*0*	*9*
*TRAUMA WARD*	*18*	*6*	*8*	*11*	*1*	*8*	*5*
*SURGICAL WARD 3*	*13*	*1*	*6*	*5*	*2*	*0*	*12*
*SURGICAL WARD 4*	*13*	*4*	*5*	*5*	*1*	*1*	*7*
*TOTAL*	66 (100 %)	*18* *(27 %)*	*27* *(41 %)*	*30* *(45 %)*	*8* *(12 %)*	*9* *(14 %)*	*41* *(62 %)*

**Fig. 5:**
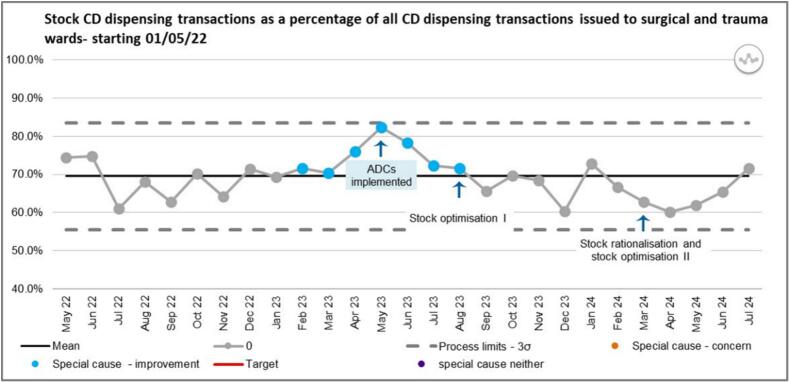
SPC showing stock CD dispensing transactions as a percentage of total number of CD dispensing transactions.

There was an increase in the number of CD stock requests expressed as a percentage of the total number of CD requests in the initial period following implementation which is likely due to the increase in CD stock requests observed following ADC implementation (Fig. 5). The ratio of stock to non-stock transactions dispensed returned to pre-implementation levels following the first stock optimisation. The number of stock CD requests declined between January and April 2024. An incline has been observed from May 2024-predictive a future trend which could be attributed to the 2nd intervention in March 2024.

**Fig. 6:**
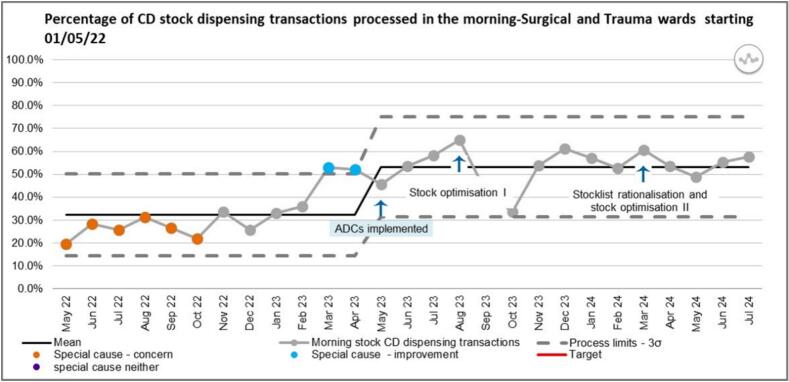
SPC showing percentage of CD stock dispensing transactions processed in the morning.

The proportion of morning stock CD dispensing workload as a percentage of the total daily CD stock workload has increased (Fig. 6). Before ADC implementation, only 32 % of the stock CD dispensing transactions were processed in the mornings. This percentage was representative of the mean calculated across the twelve-month baseline period pre-ADC implementation. The mean increased to 54 % in the period following ADC implementation (May 2023–July 2024), an improvement which was sustained apart from October 2023 where there was a decline in the volume of workload being processed in the mornings, although still within the LCL. This was caused by the implementation of a new Trust-wide EPMA system which translated to work taking longer to process thereby not being completed by midday due to the staff's unfamiliarity with the new system and general troubleshooting of system problems. The process limits were changed on the SPC due to this sustained change and the sustained improvement was shown in the SPC chart by common cause variation. There were two astronomical data points observed in March and April which coincided with the beginning of ADC implementation of the surgical and trauma wards and as such are likely to have been caused by this.

**Fig. 7:**
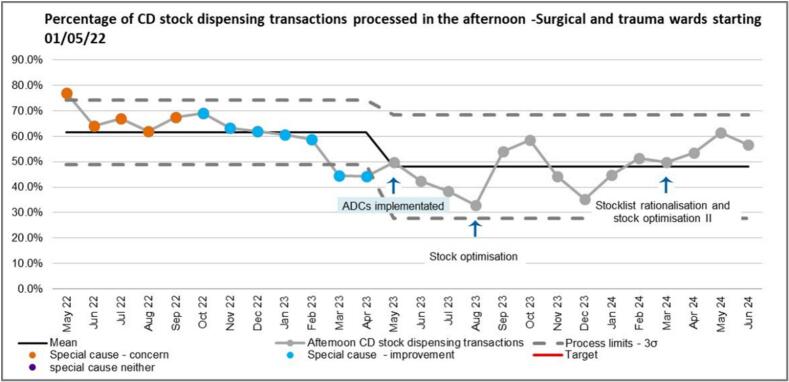
SPC showing percentage of CD stock dispensing transactions processed in the afternoon.

Afternoon stock CD workload for surgical and trauma wards started to reduce from October 2022 even before the introduction of ADCs (Fig. 7). The reasons for this were not due to any change made as part of this QI project because this occurred during the baseline period. In the baseline period before the introduction of ADCs (May 2022–April 2023), 62 % of the CD stock dispensing transactions were processed in the afternoons (mean), following the introduction of ADCs, this reduced to a mean of 48 % -an improvement which was sustained throughout the study period. The process limits were changed because of the sustained improvement and the sustained improvement was shown in the SPC by common cause variation. There was an increase in afternoon workload in October 2023 which coincided with the launch of the new Trust-wide EPMA system.

**Fig. 8:**
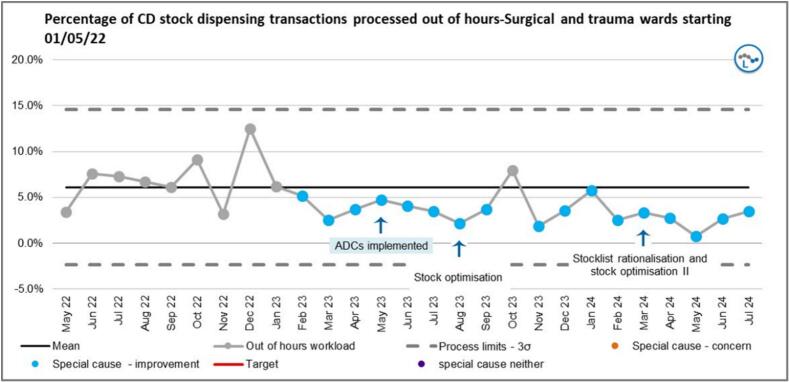
SPC showing out of hours stock CD dispensing transaction as a percentage of total CD stock workload.

Fig. 8 shows the percentage of stock CD dispensing transactions for surgical and trauma wards that were processed out of hours. A run of six points below the mean was observed from the beginning of ADC implementation (March 2023) representing a special cause improvement (a shift) until October 2023 when the new Trust-wide EPMA system was launched. Apart from this hiatus, another shift was observed from November through to July 2024 representing special cause improvement. The lowest percentage of out-of-hours dispensing transactions processed for the entire study period was observed in May 2024. This occurred two months after the second intervention.

**Fig. 9:**
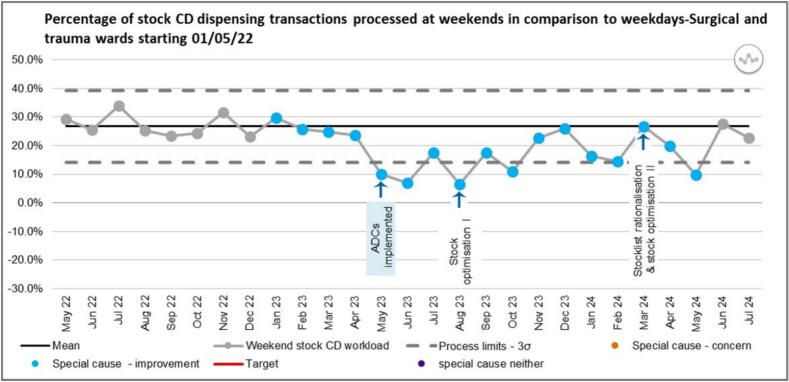
SPC showing weekend stock CD dispensing transaction as a percentage of total CD stock workload.

Fig. 9 shows the percentage of stock CD dispensing transactions for surgical and trauma wards that were processed at weekends, in comparison to weekdays. One data point falling outside the LCL has observed post-ADC implementation which was representative of a special cause improvement (astronomical point). Following the first intervention in August 2023, a run of six points below the mean (a shift) and two data points outside of the LCL (astronomical point) were observed, both representing a special cause improvement. A sustained improvement has not been observed following the second intervention although the data points sit within the control limits.

**Fig. 10:**
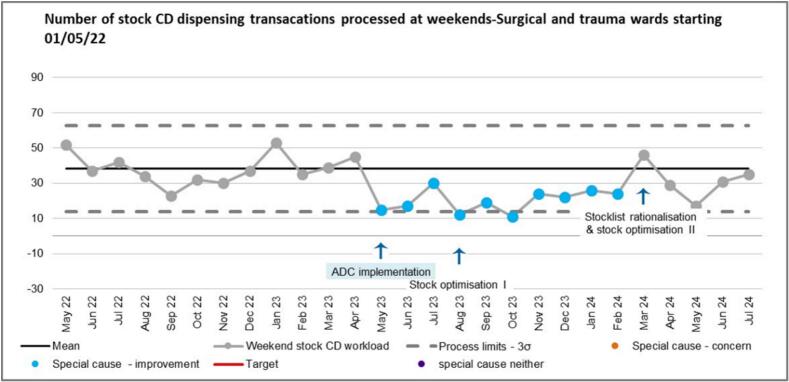
SPC showing total number of stock CD dispensing transactions at weekends.

Fig. 10 shows the volume of stock CD dispensing transactions for surgical and trauma wards that occurred at weekends. A run of more than six points below the mean was observed following the completion of ADC implementation in May 2023 representing a special cause improvement (a shift) until March 2023 when the second intervention was made; a future special cause improvement is predicted following this intervention with four data points already below the mean.

### 4.1 Lessons learned and limitations

The ability to automate the ordering and restocking process for CDs through the utilisation of ADCs not only supports CD stock inventory management in clinical locations but also ensures CD stock requests are processed earlier in the day. This helps with the dispensary workload over the course of the day and helps when it comes to the allocation of resources. The reduction in out-of-hours and weekend stock CD dispensing transactions was also noted to be significant. This result indicates that the QI set out what it aimed to achieve for this objective. The standardised formula could be adopted by other centres store CDs in ADCs or who are considering doing so. This has been proven to assist with streamlining workload via central pharmacy while ensuring sufficient stock levels are maintained to prevent patients from sustaining missed or delayed doses of their medications.

Despite the automated ordering, stock rationalisation and optimisation interventions delivered; a proportion of CD orders continue to be ordered manually by nursing staff. This means that there is still scope to further improve the standardised formula developed for this QI project to optimise stockholding. One suggestion is instead of using the total number of days over the period of data collection as the denominator, the denominator could instead be the total number of days the item was supplied to a patient. In this way, the inventory levels set would more accurately reflect the required stockholding making stockouts and therefore the requirement for manual ordering less likely. This is particularly pertinent for infrequently used stock CDs that are used in high quantities e.g. methadone liquid. Based on the formula used to work out PAR levels in this QI, if 40 mL of methadone liquid was administered to a patient twice a day for two days over three months, the daily consumption would equate to 1.78 mL/day over 90 days resulting in a PAR level of 150 mL-(1.78 mL × 7 days and rounded up to the nearest bottle size plus half a bottle). Instead, by dividing by the number of days the medication was used, a PAR level of 450 mL would be calculated (total volume used over the period/no of days used 160 mL/2 = 80 mL × 5 days). The ROL and critical levels set also need to be reviewed. In the original example, the ROL would work out to be 7.1 mL (1.78 mL × 4 days). This would mean that the system would generate an order only when the volume reached this level. In the latter example, the ROL would be set at 160 mL (daily usage X 2 days) and a critical level could be set for one-day stockholding i.e. 80 mL. Note the number of days stock holding has also been adjusted to 5, 2 and 1 respectively because the daily usage is more accurately defined using this formula and space limitations in the cabinet would preclude the inventory levels being set too high. In theory, the optimal formula would ensure stockholding levels match drug usage most of the time but there are always going to be occasions when medication use falls outside of normal patterns therefore it is impossible to mitigate for every eventuality. In addition to this, it is impossible to prevent non-stock CDs from being ordered due to the heterogeneity of patients coupled with the variation in clinicians' prescribing practices. The suggested revisions to the formula could be tested as part of a future QI.

Although not quantified, there has been feedback from the dispensary team that many of the manual orders requested by nursing were found to be a duplicate of automated CD orders generated by ADCs. This points to persisting inefficiencies in the system. Before ordering CD stock items, nurses should check the ADC to ensure the order has not already been automatically generated. This step is included in local Standard Operating Procedures (SOP) developed locally for nursing staff. A recommendation from this QI is that clinical pharmacists should screen all written orders by nursing staff to prevent duplicate orders reaching the dispensary. This practice change could be adopted by other centres who store CDs in ADCs or who are considering doing so.

The introduction of a new trust-wide EPMA system in October 2023 caused delays with the processing of workload in the dispensary. This was reflected in the SPC charts looking at the time-of-day CD workload was being processed. In October, afternoon and out-of-hours CD workloads increased whilst the morning workload was reduced. This was a direct result of the staff's lack of familiarity with the new system and identification of problems with the new system. It is important to consider the impact of any changes to existing or new EPMA systems when ADCs are profiled interfaced with the EPMA system. It is important to engage with providors well in advance of any updates or changes so that possible negative consequences of the change can be mitigated.

A potential limitation of this QI is that it was focused on surgery and trauma wards only, however this should not restrict the generalisability of results because Surgery and Trauma wards were chosen due to the variety of CDs stocked in these locations. Additionally, only one balancing measure was identified which was not sufficient. Future studies should capture the volume of CD delivery paperwork which was inappropriately returned to the pharmacy rather than being securely stored in clinical locations as part of record-keeping legal requirements. Every time this occurred, the dispensary team contacted the ward requesting for a nurse to collect the paperwork from a dispensary. An incident report was also completed each time this happened. It transpired that this occurred very regularly, especially in the period following implementation. This was extremely time-consuming for both nursing and pharmacy staff and negated some of the efficiency savings described in this project, especially in the initial stages following implementation. Hospital teams considering the use of ADCs for the storage of CDs in clinical areas should plan for this unintended consequence.

The benefits in terms of reduction in the frequency of orders and minimisation of out-of-hours and evening workload started to plateua six months after the intervention was made. Locally, this data has been used to determine the frequency of the optimisation cycles although a follow-up QI would need to be undertaken to definitively determine this. Optimisation cycles are resource intensive, especially in organisations that have a high number of ADCs. It is imperative that sufficient resource is planned for this activity which is required to be repeated cyclically.

## 5 Conclusion

Implementation of ADCs to clinical locations alone increased stock CD workload in the main dispensary in Pharmacy. A reduction in weekend and out-of-hours CD stock workload was also observed, shifting workload from afternoons to mornings for surgical and trauma wards. Stock optimisation and stock rationalisation were necessary to reduce the frequency of stock CD requests received which was achieved by aligning the PAR, re-order and critical levels to specified stockholding requirements thus ensuring the most frequently used CDs were stocked in ADCs.

This is the first study published that aimed to develop a standardised formula for setting CD stock inventory levels in ADCs to ensure sufficient stockholding whilst controlling the frequency of orders generated to the central pharmacy thus streamlining dispensary workload by directing work towards weekday mornings and away from afternoons, out of hours and weekends. Stocklist optimisation and stocklist rationalisation; both interventions used in this QI, should be periodically undertaken as this is a continuous cycle of improvement which is influenced by many external factors for example prescribing preference and patient heterogeneity. The SPC chart data suggests that the frequency of stock optimisation and stock rationalisation should be undertaken at least every six months.

The proposed improvements to the standardised formula incorporating more than one stock optimisation and rationalisation cycle is recommended as the next steps to this QI.

## CRediT authorship contribution statement

**Áine Walsh:** Writing – review & editing, Writing – original draft, Visualization, Validation, Methodology, Formal analysis, Data curation, Conceptualization. **Emma Jeffrey:** Writing – review & editing, Project administration. **Kit Lai:** Writing – review & editing, Validation, Methodology, Investigation, Conceptualization.

## Declaration of competing interest

The authors declare that they have no known competing financial interests or personal relationships that could have appeared to influence the work reported in this paper.
